# Massively Sub-wavelength Guiding of Electromagnetic Waves

**DOI:** 10.1038/srep07495

**Published:** 2014-12-16

**Authors:** I. R. Hooper, B. Tremain, J. A. Dockrey, A. P. Hibbins

**Affiliations:** 1 Department of Physics and Astronomy, University of Exeter, Stocker Road, Exeter, EX4 4QL

## Abstract

Recently a new form of ultra-thin flexible waveguide consisting of a conducting comb-like structure with a thickness of the order of 1/600*^th^* of the operating wavelength was presented. However, whilst the thickness of the guide was massively sub-wavelength, the remaining dimensions (the height and period of the comb) were much longer. In this paper we propose, and experimentally verify, that a modified guiding geometry consisting of a chain of ultra-thin conducting spirals allows guiding of electromagnetic waves with wavelengths that are many times (40+) longer than any characteristic dimension of the guide, enabling super-sub-wavelength guiding and localisation of electromagnetic energy.

The control of light, and of electromagnetic fields in general, has been a topic of interest throughout the history of science, from the simple refractive and reflective instruments of early optics, to more advanced schemes such as the recent developments in the field of transformation optics^1^,^2^. One aspect of this control that has evolved greatly over the last century is the channeling, or guiding, of light in various forms of waveguides such as fibre-optics, hollow metallic guides, various forms of transmission lines, and surface-waves; descriptions of all of which can be found in most good electromagnetic text books^3^. In this paper we will demonstrate an extreme form of sub-wavelength waveguide formed from a chain of connected conducting spirals and show that it can support bound guided waves with wavelengths many times longer than any characteristic dimension of the structure.

We will begin by considering a well-known form of surface-wave waveguide, that of a conducting surface formed from an array of infinitely long rectangular profile protrusions^4^. The geometrical structuring creates an artificial electromagnetic boundary condition at the top surface of the dominos due to the penetration of the electromagnetic fields in to the domino cavities, and this artificial boundary condition is of the form that allows a bound surface wave to be supported. The dispersion relation of this surface wave is similar in form to that of the well-known surface plasmon polariton (SPP)^5^ and asymptotically approaches the frequency at which the cavities support a quarter-wavelength resonance. Such artificially textured guiding structures were well-known in the field of electrical engineering in the mid 20th century^6^, but have seen a resurgence of interest in recent years in the physics community after being rediscovered and termed “spoof” or “designer” SPPs^7^,^8^. Indeed, it should be noted that there are a wide range of artificially structured surfaces that can support similar bound modes; the essential requirement being that the textured surface must exhibit an electromagnetic resonance due to its geometry, and one can envisage infinite variation on this theme.

Recently, Martin–Cano et al.^9^ predicted that the lateral dimension of these rectangular profiled surfaces could be narrowed without significantly perturbing the character of the guided wave, and they termed this narrowed structure a “domino array”, and the supported modes as “domino plasmons” (see figure 1). This insensitivity to the lateral dimension, *t*, results from the magnetic field within the cavities remaining entirely transverse regardless of the value of *t* and, since there is no additional quantisation of the magnetic field, the resulting dispersion relation remains relatively unperturbed. Full discussions of this can be found in refs 9,10,11.

Subsequent to Martin–Cano et al.'s work, Brock et al. observed these modes experimentally^10^, and Ma et al. fabricated power dividers, directional couplers, and ring resonators using appropriately designed domino-based geometries^12^. Shen et al.^11^ then took the idea to the extreme and made the lateral dimension of the dominos massively sub-wavelength, forming a flexible waveguide. They did this by using standard etching techniques to form arrays of dominos that were only 18 *μ*m in thickness on flexible polymer substrates (see figure 1), and demonstrated guiding of waves with free-space wavelengths of 25 mm (in other words, the operating wavelength was approximately 1400 times the lateral width of the dominos, and 600 times the thickness including the substrate). They referred to these modes as “Conformal Surface Plasmons” or CSPs, and demonstrated the flexibility of their structures by coiling the waveguide into complex geometries. Following this, the same group demonstrated a dual-band waveguide formed from arrays consisting of two sizes of dominos^13^, and Liu et al.^14^ investigated the higher order modes of similar ultra-thin domino geometries (the guided modes corresponding to multiples of the fundamental quarter wavelength resonance of the domino cavities). We should also note that other thin metamaterial geometries can also act as waveguides such as lines of split-ring resonators that support and guide magneto-inductive waves^15^,^16^, and chains of plasmonic particles at optical frequencies^17^,^18^. However, it should be noted that all these systems rely upon the same basic principle: the guided modes arise from the coupling between the individual localised resonators.

These CSP based geometries are an example of an “open-waveguide” whereby the guided fields are open to the bounding dielectric (as opposed to closed waveguides such as coaxial cables). Whilst closed waveguide geometries have huge advantages for many applications (for example a piece of coaxial cable can guide radio frequency waves within a volume many times smaller than it's wavelength) there are some applications for which they are unsuited and for which open waveguides may be better candidates. For example, CSP geometries have been suggested as interconnects for THz circuitry due to their planar sub-wavelength nature, and the ease with which they may be coupled to using standard transmission line technologies^19^. In addition, for on-body sensors^20^, and, more generally, any sensing application in which the guide itself may be used as the sensing transducer through interactions of the guided fields with the local environment, open waveguides such as those based on CSPs may be a natural choice.

Whilst much of the previous work on dominos geometries has been undertaken on structures that are massively sub-wavelength in a single dimension, the height, *h*, of the dominos has remained of the order of *λ*_0_/4 since the mode is only highly confined at frequencies close to the asymptotic limit determined by the geometrical resonance of the domino cavities. However, one might wish to reduce the overall dimensions of a waveguide due to, for example, their potential use as interconnects in photonic circuits, or because of the general interest in concentrating electromagnetic energy into sub-wavelength volumes. In order to achieve this one requires a design that exhibits a geometrical resonance at a wavelength that is much longer than any overall dimension of the geometry. This is, in fact, rather simple to achieve using spiral cavities, which can have cavity lengths many times longer than their geometrical size. Indeed, one might envisage an array of spiral cavities as an array of tall thin domino cavities that have subsequently been “rolled up” and, since the spiral cavity array would thus be topologically similar to the domino array, one might expect them to exhibit the same insensitivity to the lateral dimension (see figure 1). We also note that individual thin metallic spiral geometries are of significant interest in their own right having recently been shown to support “spoof” localised surface plasmon modes with both electric and magnetic dipolar field configurations^21^.

## Results and Discussion

**Figure 2:**
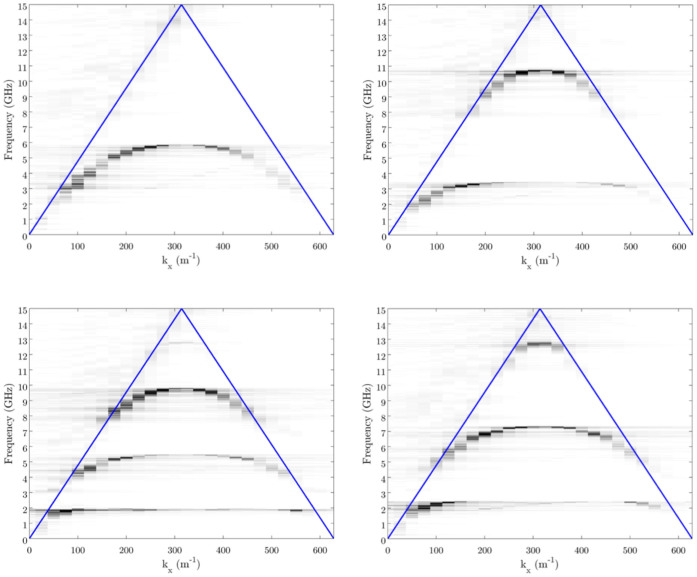
The Fourier amplitude as a function of frequency and wavevector along the chain of spirals for geometries with different numbers of vertices. The dispersion relations of the guided modes supported by these ultra-thin connected spiral geometries are clearly evident. Also shown on the plots as blue lines are the scattered and unscattered light lines. Clockwise from top-left: 1, 3, 5 and 7 vertices.

In figure 2 we present the Fourier magnitude as a function of wavevector and frequency for connected spiral geometries consisting of 1, 3, 5 & 7 vertices corresponding to increasingly tightly wound spirals (see methods section), and the dispersion curves of the guided modes supported by the geometries are clearly evident as dark bands. Also shown in figure 2 are the un-scattered and scattered light lines (originating from the origin and from *k_x_* = 2*π*/*λ_g_*, where *λ_g_* is the periodicity of the spirals, respectively). Note that the dark bands exist only in the non-radiative region outside of the un-scattered lightline (they have greater in-plane wavevectors than those available to freely propagating waves) indicating that they are bound guided modes propagating along the spiral chain. It is also clear from figure 2 that, as the number of vertices in the spirals is increased, the high frequency limit of the lowest order mode decreases, and higher order guided modes become supported. This can be easily understood by considering the cavity lengths, and resonant frequencies, of the spiral geometries.

For square spiral geometries with odd numbers of vertices the cavity length, *l*, can be determined using,


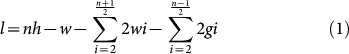


where *h* is the height (and pitch) of the square profiled spiral geometry, *g* is the width of the air gaps, *w* is the line width, and *n* is the number of vertices (see figure 4). For a constant line width, as used here, the width of the air gaps is given by:


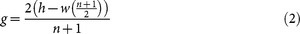


From knowledge of the cavity lengths it is a simple matter to predict the resonant frequencies of the cavities, and hence the high frequency cut-offs (the asymptotic limits) of the guided waves,


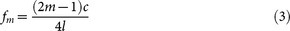


where *f_m_* is the resonant frequency of the *n^th^* order cavity mode and *c* is the speed of light in vacuum.

Using eqns. 1 & 2 we obtain asymptotic limits for the fundamental modes of our spiral chains with 1, 3, 5 & 7 vertices of 7.77 GHz, 3.96 GHz, 2.65 GHz & 2 GHz, respectively. The geometry with only a single vertex is similar to that of the original domino array studied by Shen et al., though with a much smaller line width, and from figure 2 we can clearly observe the asymptotic limit at approximately 6 GHz. This is significantly lower than the predicted limit of 7.77 GHz. It is noticeable that the comparison between the predicted asymptotic limits and those determined from the measurements improves as the number of vertices, and hence the cavity lengths, increases. This is due to diffractive effects, arising from the periodic nature of the waveguide, becoming less pronounced when the operating wavelength is much greater than the periodicity. Since the periodic nature of the system requires that the dispersion curves be flat-banded at the Brillouin zone boundary, a decrease in the high frequency limit of the bands must occur as a result of the periodicity. However, if the operating wavelength is much greater than the periodicity, the dispersion curves of the supported modes are already flat-banded at the Brillouin zone boundary and any modification of the high frequency limit will be small. Thus the simple analytical model based on the cavity length alone more accurately approximates the measured asymptotic limits for the lower frequency modes.

**Figure 3:**
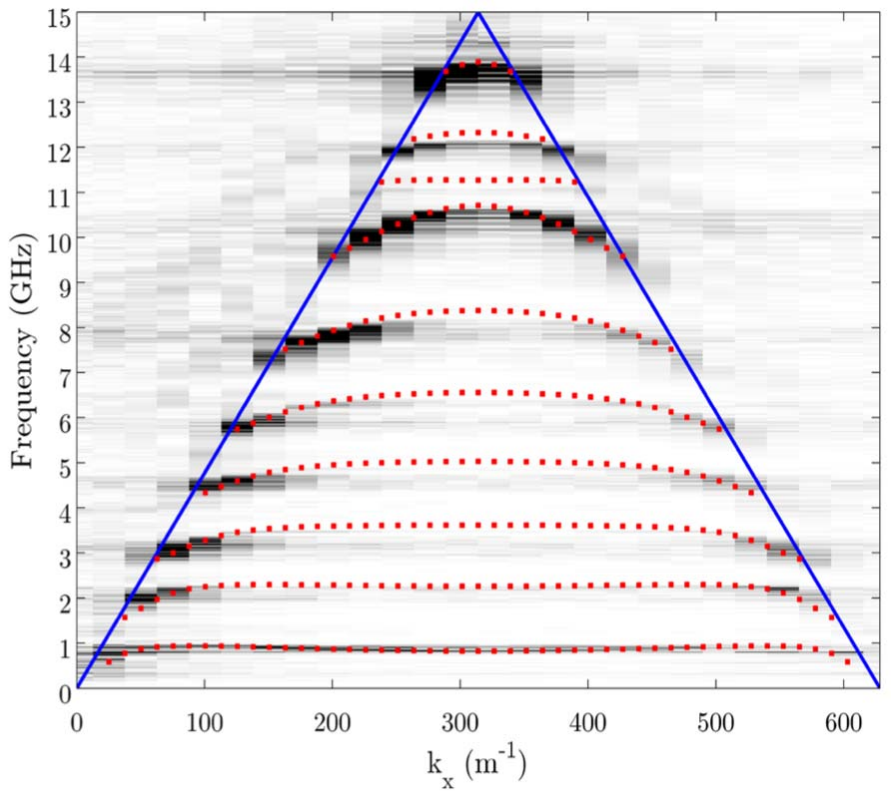
The Fourier amplitude as a function of frequency and wavevector along the chain of spirals for the connected spiral chain geometry with 19 vertices. The red dots are the mode positions as calculated using the eigensolver in Comsol Multiphysics.

**Figure 4:**
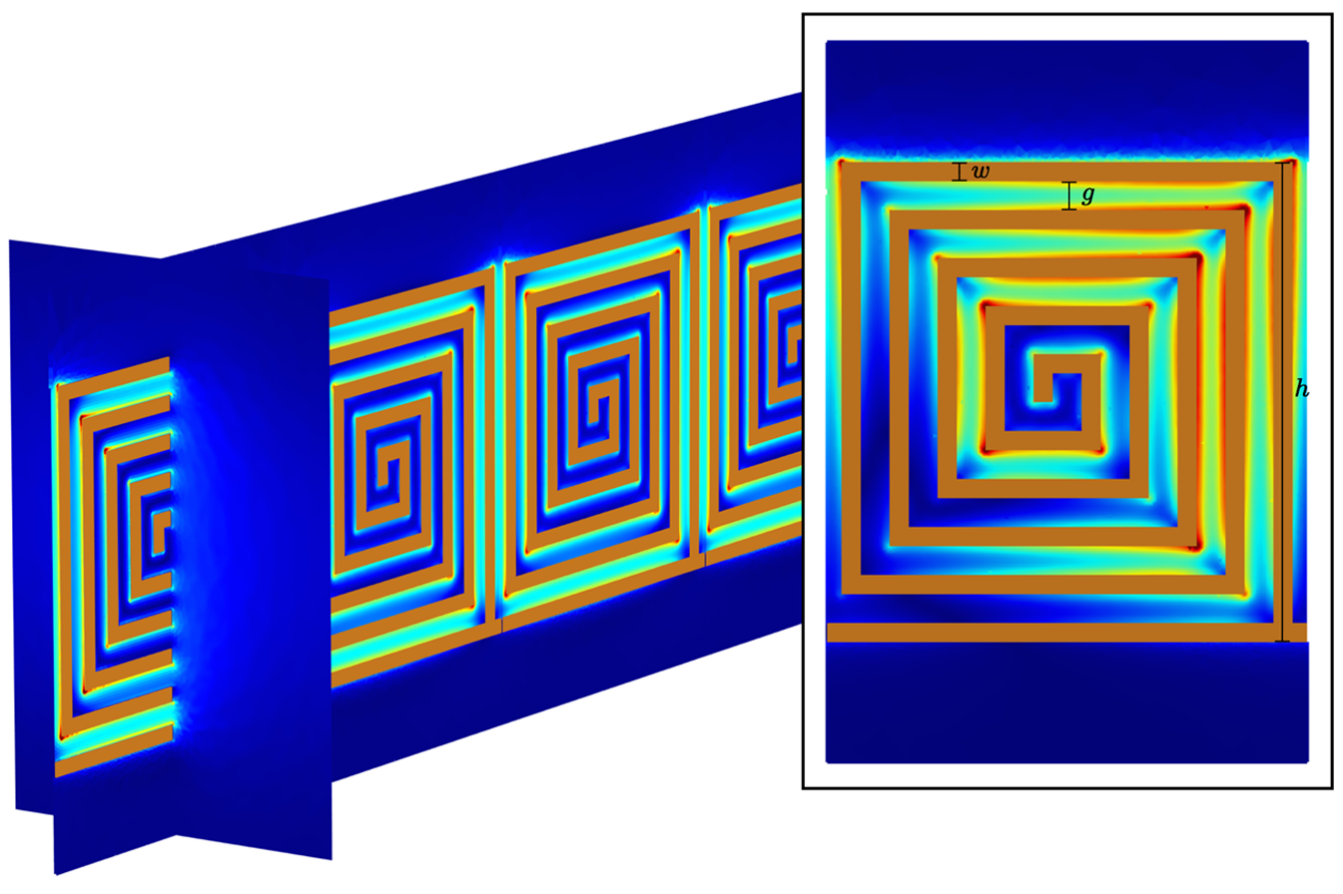
Modelled electric field norm profile of the fundamental guided mode at *k_x_* = *π*/*λ_g_* for the system described in figure 3. Inset: The same but for the 4th order mode. Blue corresponds to low field strengths and red to high field strengths.

In figure 3 we show the Fourier amplitude as a function of wavevector and frequency for the most dense spiral geometry studied, one consisting of 19 vertices. The path length of this spiral geometry obtained using eqns 1 & 2 is almost 10 cm in a spiral with a geometrical size of only 1 cm. With this path length the expected resonant frequency of the fundamental cavity mode would be 0.75 GHz, which agrees extremely well with the measured value. This frequency corresponds to a free space wavelength of 40 cm; 40 times longer than the longest dimension of the spiral geometry. Also shown in figure 4 are numerically modelled predictions obtained using the eigensolver of a commercially available finite element method package (Comsol Multiphysics), which compare well with the measurements.

Finally, in order to confirm the origin of the waveguide modes as arising from coupling between the cavity modes of individual spirals we have calculated the electric field norm profiles at the Brillouin Zone boundary (*k_x_* = *π*/*λ_g_*) for the first and fourth order eigenmodes of the numerical solutions shown in figure 3, and these are shown in figure 4. For the fundamental mode there exists only a single node in the electric field profile, which occurs at the center of the spiral. This is similar to the fundamental mode of the domino cavity which exhibits a single node at the base of the cavity. For the fourth order mode, however, an additional three nodes exist, demonstrating that this guided mode is associated with the fourth order cavity mode of the spirals. The extreme confinement of the fields to the region around the spirals is also clearly evident and we can determine their extent in the transverse direction (the distance over which the field has decayed to 1/*e* of its maximum value) using *L_T_* = 1/*k_T_*, where *k_T_* is the transverse wavenumber given by 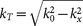. This gives a value of 3.3 mm, or 1/120*^th^* of the free-space wavelength, for the fundamental mode at the Brillouin Zone boundary.

We have demonstrated that a connected chain of ultra-thin spiral cavities can act as a massively sub-wavelength flexible waveguide for electromagnetic waves. The operating wavelength of these waveguides is determined by the length of the spiral cavities, which can be very long relative to their geometrical size. Indeed, for the fundamental waveguide mode of the longest spiral cavity studied here the operating wavelength of the guide is approximately 40 times the longest dimension of the geometry. It should be noted, however, that more tightly wound spirals, with corresponding longer cavity lengths, could support modes with even longer wavelengths. These massively sub-wavelength waveguides may be attractive candidates as interconnects in photonic circuits, or as a means to concentrate electromagnetic energy into extremely sub-wavelength volumes.

## Methods

**Figure 5:**
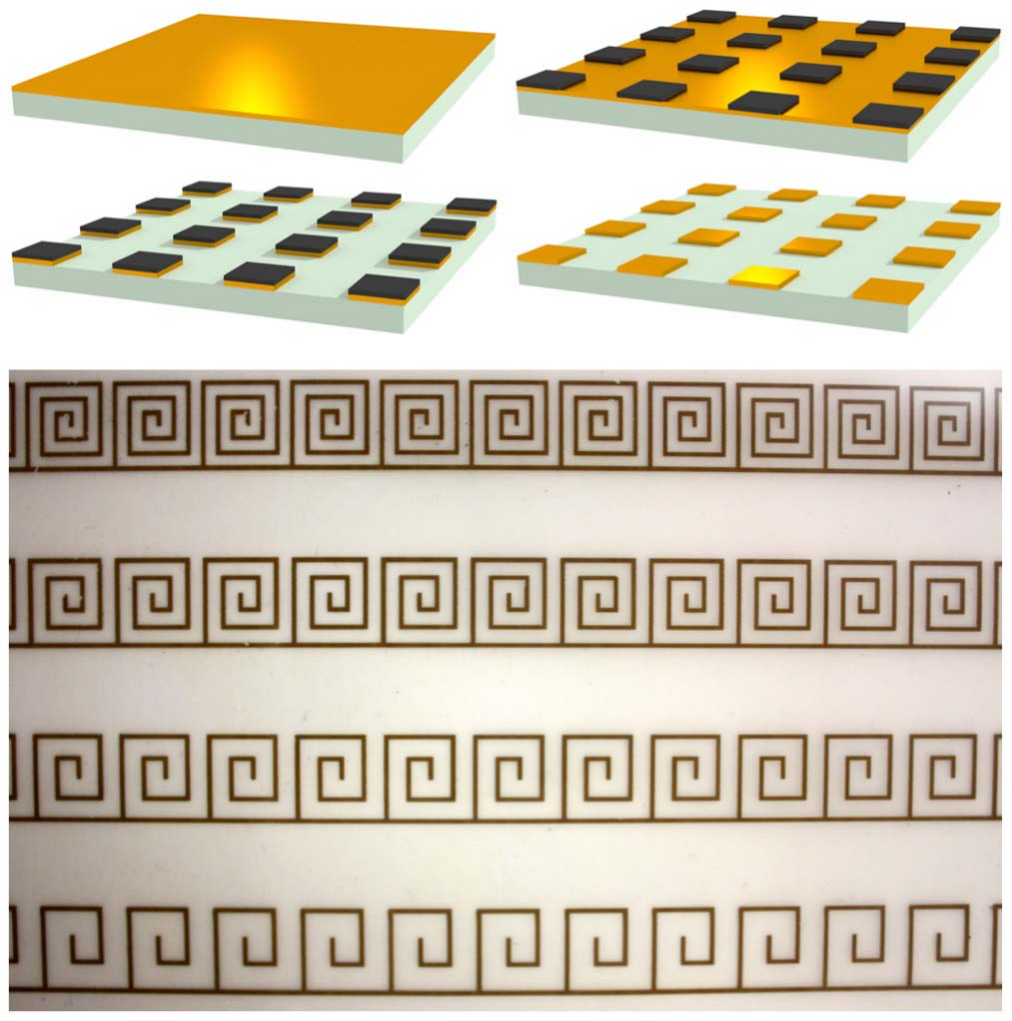
Top: A schematic showing the lithographic fabrication method. A 50 *μ*m thick polyester substrate coated with 18 *μ*m of copper has the desired pattern printed on to it using a solid ink printer (laser printers can also be used). The printed pattern forms an etch mask, and the unmasked copper is removed using ferric chloride. The ink mask is subsequently removed by solvent assisted abrasion. Bottom: Example of the fabricated samples showing spiral designs with 5, 7, 9 and 11 vertices.

The chains of connected spirals with different cavity lengths were fabricated using a standard lithographic technique. A 50 *μ*m thick flexible sheet of polyester coated with 18 *μ*m of electrodeposited copper (available from GTS Flexible, prod. no.: 550920ED) had the required design printed on to it using a commercially available solid ink printer (Xerox ColorQube 9301). The printed ink design acts as an etch mask when the sample is placed into a ferric chloride etch solution, which removes any un-masked copper. The remaining solid ink is subsequently removed using solvent assisted abrasion. Ten designs of square profiled spiral cavity chains were fabricated, with each design being specified by the number of vertices in the spiral. Designs with 1, 3, 5…19 vertices were fabricated, and the line width of the copper tracks (350 *μ*m), and the height (*h*) and periodicity (or pitch, *λ_g_*) of the spiral array (10 mm), were kept constant such that the cavity length of each spiral increased with the number of vertices (see figure 5).

The guided waves supported by the spiral chain were excited using a near-field source (a 2 mm section of stripped coaxial cable driven by the source port of a vector network analyser (VNA)). The instantaneous electric field strength was subsequently measured as a function of distance along the midline of the array approximately 3 mm from the surface using a stripped coaxial probe connected to a second port on the same VNA. By performing a Fast Fourier Transform (FFT) on the resulting spatially dependent field profile the wavevectors of any excited guided waves becomes clearly evident as peaks in the Fourier spectrum, and by undertaking this process for a range of frequencies the dispersion curves of the excited modes were determined.

## Figures and Tables

**Figure 1:**
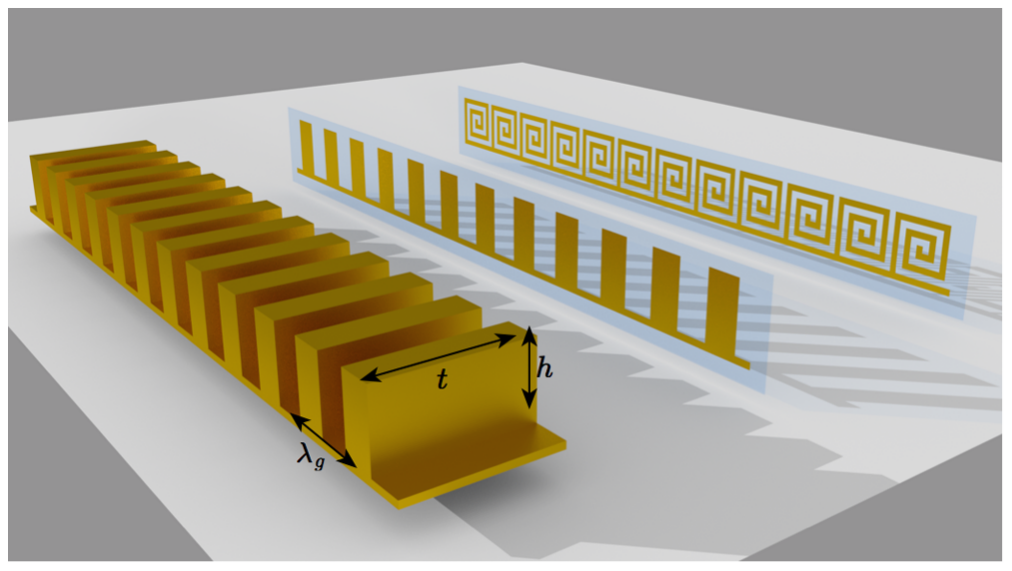
The three geometries discussed. Left: a domino array consisting of a periodic array of conducting slabs on a conducting ground plane. Middle: An ultra-thin domino array on a flexible polymer substrate. Right: A chain of ultra-thin connected conducting spirals on a flexible polymer substrate.
